# Vitamin D_3_ as an immunomodulatory agent: molecular mechanisms, clinical translation, and precision therapeutic strategies

**DOI:** 10.3389/fimmu.2026.1770141

**Published:** 2026-03-02

**Authors:** Qing Liu, Zhenzi Li, Shaojie Li, Yue Li, Haifeng Pan, Ye Tao

**Affiliations:** 1 Department of Otolaryngology-Head and Neck Surgery, The First Affiliated Hospital of Anhui Medical University, Hefei, Anhui, China; 2 Department of Epidemiology and Biostatistics, Anhui Medical University, Hefei, Anhui, China

**Keywords:** autoimmune diseases, drug delivery systems, immunomodulation, inflammation regulation, precision medicine, VDR signaling, vitamin D_3_

## Abstract

Vitamin D_3_ (VitD_3_) deficiency affects over one billion individuals globally, representing a critical modifiable risk factor for immune-mediated diseases. Beyond its classical role in calcium metabolism, Vitamin D_3_ orchestrates immune homeostasis through vitamin D receptor (VDR) signaling, exerting profound regulatory effects on both innate and adaptive immunity. Mechanistically, Vitamin D_3_ maintains the balance between antimicrobial defense and inflammatory suppression by inhibiting key pro-inflammatory pathways including nuclear factor κB (NF-κB) and the NOD-like receptor protein 3 (NLRP3) inflammasome, while activating the Nuclear Factor Erythroid 2-Related Factor 2 (Nrf2)-mediated antioxidant defense system. However, the immunomodulatory effects of Vitamin D_3_ exhibit significant inter-individual variability, with clinical efficacy highly dependent on patient-specific factors including serum 25-hydroxyvitamin D [25(OH)D, calcifediol] levels and *VDR* gene polymorphisms, driving a paradigm shift from empirical supplementation toward biomarker-guided precision medicine. Novel delivery systems—nanoemulsions, twin-screw extrusion technology, and liposomes—effectively overcome bioavailability and stability limitations of traditional preparations. This review systematically examines the immunomodulatory mechanisms of Vitamin D_3_, evaluates clinical translation evidence in psoriasis, systemic lupus erythematosus (SLE), rheumatoid arthritis (RA), type 1 diabetes mellitus (T1DM), inflammatory bowel disease (IBD), and discusses precision medicine strategies and therapeutic potential.

## Graphical Abstract

**Figure:**
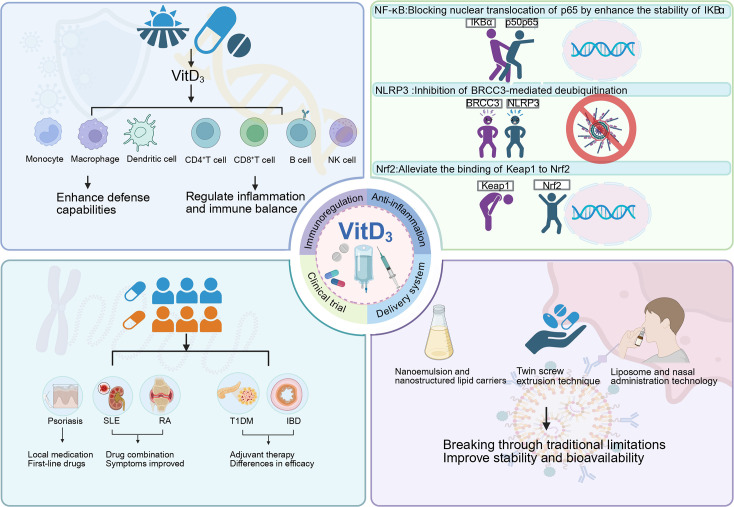

## 1 Introduction

Vitamin D_3_ deficiency constitutes a critical global public health crisis affecting over one billion individuals worldwide, with particularly high prevalence among pregnant women, obese populations, and the elderly (1). Vitamin D_3_ serves as a pivotal nexus bridging the endocrine and immune systems, with functions extending far beyond its classical role in calcium-phosphorus homeostasis. Two decades of rigorous research have definitively established that immune cells not only express Vitamin D receptor (VDR) but also possess the complete enzymatic machinery required for local Vitamin D_3_ metabolism activation (2). Epidemiological studies consistently demonstrate inverse correlations between serum 25-hydroxyvitamin D [25(OH)D, calcifediol] concentrations and the incidence of various immune-mediated diseases, including systemic lupus erythematosus, rheumatoid arthritis and so on (3–5). Relevant research indicates that Vitamin D_3_ supplementation may significantly prevent the development of autoimmune diseases (6, 7). This conclusion gains further support from latitudinal gradient studies demonstrating higher disease incidence in high-latitude regions with reduced ultraviolet B (UVB) exposure (8).

Vitamin D_3_ is a nutrient rather than a drug, obtainable through diet and sunlight exposure, making it inherently difficult to establish a true “zero-exposure” placebo group in clinical trials. Background Vitamin D_3_ levels shared by both intervention and control groups dilute the supplementation effect, biasing results toward the null. This may explain why multiple large-scale randomized controlled trials have failed to demonstrate significant preventive effects of Vitamin D_3_ supplementation on autoimmune disease incidence (9, 10), revealing the inherent complexity of the Vitamin D_3_–immune function relationship. Moreover, Confounding factors including genetics, diet, and infections further complicate the interpretation of research findings (11–13).

Given that genetic factors contribute approximately 65% of individual Vitamin D_3_ level variance, precision medicine approaches become increasingly important (14). Through biomarker-guided patient stratification based on serum 25(OH)D levels and *VDR* gene polymorphisms, treatment responders can be precisely identified across different disease phenotypes, genetic backgrounds, and baseline states, thereby overcoming the limitations of conventional “one-size-fits-all” supplementation approaches, maximizing therapeutic benefits while minimizing resource waste. Meanwhile, traditional Vitamin D_3_ preparations are limited in clinical application due to low bioavailability and poor stability (15). Advances in novel delivery systems—including nanoemulsions, twin-screw extrusion technology, and liposomal formulations—provide effective solutions to these problems by enhancing intestinal absorption, improving stability, and enabling targeted delivery to optimize the pharmacokinetic properties of Vitamin D_3_ (15, 16).

Vitamin D_3_ immunology is a rapidly progressing research field. Here, we provide a comprehensive review of the immunomodulatory mechanisms of Vitamin D_3_, its pathological roles of immune system diseases, and discuss its therapeutic potential and precision medicine strategies. The schematic overview illustrating the core themes and translational framework of this review is presented in the **Graphical Abstract**.

## 2 Vitamin D_3_ metabolism and VDR signaling

**Figure 1:**
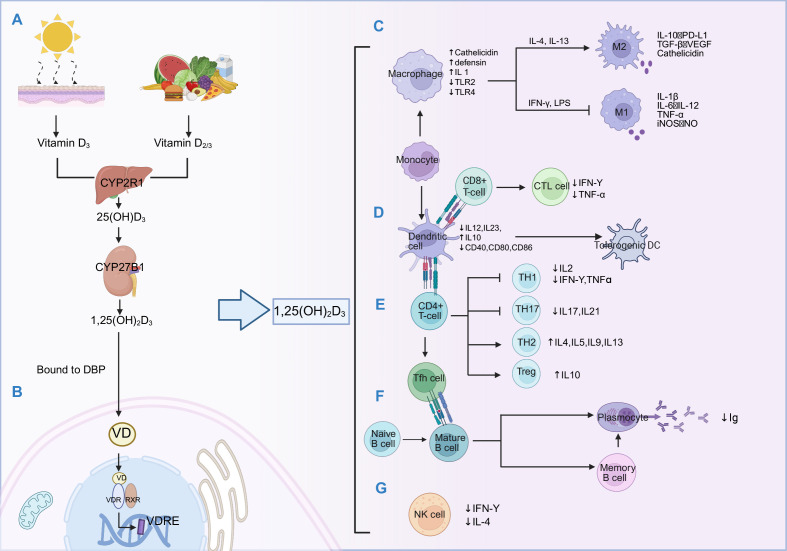
Vitamin D_3_ metabolism and its mechanism in the immune system. **(A)** Metabolic pathway: Following cutaneous synthesis or dietary intake, Vitamin D_3_ is hydroxylated to 25(OH)D_3_ by hepatic CYP2R1, then converted to the active form 1,25(OH)_2_D_3_ by CYP27B1 in the kidney or immune tissues. **(B)** VDR signaling: 1,25(OH)_2_D_3_ binds to VDR to form VDR-RXR heterodimer that bind to VDREs to regulate target. **(C)** Macrophage regulation: Vitamin D_3_ upregulates phagocytic receptors (CD14, CRIg) via ERK1/2, p38 MAPK, and JNK pathways (196), induces M2 polarization, and enhances antimicrobial peptide expression (Cathelicidin, Defensin), promoting phagocytosis and autophagy (33). **(D)** DC regulation: Vitamin D_3_ suppresses NF-κB signaling to inhibit the expression of mature related molecule LAMP3 (197), and reduce maturation marker CD83, downregulates costimulatory molecules CD40, CD80, CD86, decreases IL-12 production, and induces tolerogenic DC phenotype (34); CD8^+^ T cell: downregulates perforin and granzyme B expression in CTLs, attenuating cytotoxicity (43) **(E)** T cell regulation: Regulate CD4 ^+^ T cell differentiation, inhibit Th1 (down-regulate T-bet, reduce IFN-γ, TNF-α) and Th17 (40, 41) (down-regulate RORγt, reduce IL-17, IL-21)promote Th2 (up-regulate GATA-3, increase IL-4, IL-5) and Treg (up-regulate Foxp3, increase IL-10) (42); **(F)** B cell regulation: Vitamin D_3_ inhibits B cell maturation, differentiation, and antibody secretion by reducing Tfh cell IL-21 secretion and downregulating CXCL13 expression (198); concurrently induces Breg IL-10 production to maintain immune tolerance. **(G)** NK cell regulation: reduced IFN-γ secretion and cytotoxicity of NK cells (36).

The biological activity of Vitamin D_3_ depends on a sophisticated metabolic cascade that transforms the parent compound into its hormonally active form. When UVB radiation converts 7-dehydrocholesterol to preVitamin D_3_, this intermediate undergoes thermal isomerization to form Vitamin D_3_, which subsequently binds to vitamin D binding protein (DBP) for systemic transport (17). In the liver, Vitamin D_3_ is 25-hydroxylated primarily by Cytochrome P450 Family 2 Subfamily R Member 1(*CYP2R1*), generating 25(OH)D_3_ (18) — the major circulating form and principal clinical biomarker of Vitamin D_3_ status. Subsequently, 25(OH)D_3_ undergoes 1α-hydroxylation by Cytochrome P450 Family 27 Subfamily B Member 1 (*CYP27B1*) (predominantly in renal proximal tubule cells but also locally in immune tissues) to produce 1,25-DihydroxyVitamin D_3_ [1,25(OH)_2_D_3_, calcitriol], the most biologically active metabolite (2, 19) (**Figure 1A****).** In the kidney, 1,25(OH)_2_D_3_ synthesis is tightly regulated to maintain calcium-phosphate homeostasis: fibroblast growth factor 23 (FGF23) and elevated calcium levels inhibit *CYP27B1* activity while simultaneously inducing Cytochrome P450 Family 24 Subfamily A Member 1 (*CYP24A1*) expression, the latter being responsible for catabolizing active metabolites (20, 21). Activated immune cells—including macrophages and dendritic cells (DCs)—also express *CYP27B1* and can locally generate 1,25(OH)_2_D_3_, which functions in an autocrine manner to regulate their differentiation and functional responses (22, 23). Unlike renal synthesis, immune cell *CYP27B1* is not regulated by FGF23, as these cells lack alpha-klotho (a transmembrane protein that serves as an essential co-receptor for FGF23 signaling), and is instead governed by inflammatory signals. The biological effects of 1,25(OH)_2_D_3_ are mediated through VDR, a member of the nuclear receptor superfamily. Upon ligand binding, VDR forms heterodimers with retinoid X receptor (RXR), creating complexes that bind to Vitamin D response elements (VDREs) (24)(**Figure 1B**), and recruit coactivator complexes to initiate gene transcription (classical pathway). Additionally, VDR-RXR signaling can repress gene transcription in a gene-specific manner by recruiting corepressor complexes, or through VDRE-independent mechanisms such as protein-protein interactions with key inflammatory transcription factors including NF-κB (25) and NFAT (nuclear factor of activated T cells) (26), thereby antagonizing their function and regulating genes involved in calcium homeostasis, cell proliferation, differentiation, and immunomodulation. During infection, Toll-like receptor (TLR) 25(OH)D_3_ activation in macrophages simultaneously upregulates both *CYP27B1* and *VDR* expression, significantly enhancing local 1,25(OH)_2_D_3_ production and amplifying VDR-mediated gene regulation (27). This autocrine/paracrine signaling mechanism enables immune cells to generate high local 1,25(OH)_2_D_3_ concentrations even at low systemic levels, highlighting the critical importance of tissue-level Vitamin D_3_ sufficiency for maintaining optimal immune function. Whether systemic high-dose supplementation can effectively replicate these local effects remains an unresolved question that likely depends on individual genetic characteristics and disease context.

## 3 Immunomodulatory mechanisms of Vitamin D_3_

### 3.1 Innate immune modulation

1,25(OH)_2_D_3_ plays a pivotal role in orchestrating innate immune responses, enhancing antimicrobial defense while preventing excessive inflammatory reactions that could lead to tissue damage (**Table 1**). Its typical mechanism involves VDR-mediated transcriptional activation of the Cathelicidin Antimicrobial Peptide (CAMP) gene, inducing the production of antimicrobial peptides, particularly cathelicidin LL-37 (28). Additionally, 1,25(OH)_2_D_3_ induces expression of nucleotide-binding oligomerization domain 2 (NOD2), which upon activation by bacterial muramyl dipeptide stimulates NF-κB signaling and subsequently induces human β-defensin 2 (*DEFB2*/HBD2) expression, establishing Vitamin D_3_ as both a direct and indirect regulator of antimicrobial peptide production (29, 30). 1,25(OH)_2_D_3_ modulates the phenotype and function of monocyte and macrophage through multiple mechanisms: suppressing pro-inflammatory cytokine expression, including tumor necrosis factor (TNF)-α, interleukin (IL)-1β, IL-6, upregulating negative regulators such as mitogen-activated protein kinase (MAPK) phosphatase-1 (31), and modulating microRNA expression profiles (32). Notably, Vitamin D_3_ promotes the anti-inflammatory M2-like macrophage polarization through Peroxisome Proliferator−Activated Receptor γ (PPAR γ) signaling pathways (33) (**Figure 1C**). In dendritic cells, 1,25(OH)_2_D_3_ inhibits maturation processes and arrests developmental progression while inducing tolerogenic phenotypic characteristics (**Figure 1D**). This manifests specifically as downregulation of major histocompatibility complex class II molecules (MHC-II) and costimulatory molecules (CD40, CD80, CD86) (34), enhanced IL-10 secretion, and upregulation of inhibitory molecules including Programmed Death-Ligand 1(PD-L1) (35). Furthermore, Vitamin D_3_ attenuates natural killer cell(NK cell) activity, effectively suppressing IFN-γ production while reducing both cytotoxicity and lytic function (36) (**Figure 1G**). Clinical studies and *in vitro* experiments demonstrate that Vitamin D_3_ supplementation increases antimicrobial peptide expression in peripheral blood mononuclear cells (PBMCs) (37) and reduces susceptibility to respiratory infections (38), though the magnitude of effect varies considerably with baseline Vitamin D_3_ status, seasonal factors, and individual genetic variation (39).

**Table 1:** Immunomodulatory effects of Vitamin D_3_ on major immune cells.

Types of immunocytes	Key molecules/pathways	Regulation mechanism of Vitamin D_3_	Functional result
Macrophage	VDR, MKP-1, SOCS1	Inhibit M1 pro-inflammatory phenotype, induce M2 polarization (33), enhance phagocytosis and autophagy function.	Anti-inflammatory, repair
Dendritic cell	MHC-II, CD80/86, IDO	Inhibit maturation and antigen presentation, induce tolerant DCs (34).	Reduce the inflammatory activation of T cells
CD4^+^ T cells	T-bet, RORγt, Foxp3	Inhibit Th1/Th17 differentiation and promote Treg/Th2 polarization (40, 41).	Maintaining immune tolerance
CD8^+^ T cells	Perforin, Granzyme B	Down-regulate the expression of cytotoxic factors (43).	Reduce tissue damage
B cells	NF-κB, XBP1, Cyp24	Inhibit antibody production and plasma cell differentiation, induce apoptosis (40, 45).	Reduce the burden of autoantibodies

### 3.2 Adaptive immune modulation

1,25(OH)_2_D_3_ exerts comprehensive regulatory effects on adaptive immunity, generally promoting anti-inflammatory and tolerogenic responses. During CD4^+^ T helper cell (Th) differentiation, it selectively inhibits Th1 and Th17 lineage development while promoting regulatory T cell (Treg) formation and, to a lesser extent, supporting Th2 responses (**Figure 1E**). Mechanistically (**Table 1**), 1,25(OH)_2_D_3_ downregulates master transcription factors driving Th1 (40) (T-bet, T-box expressed in T cells) and Th17 (41) (ROR-γt, RAR-related Orphan Receptor Gamma t) differentiation while simultaneously enhancing Forkhead Box P3 (FoxP3) expression in developing Treg cells (42). CD8^+^ cytotoxic T lymphocytes (CTL) are similarly subject to 1,25(OH)_2_D_3_ regulation: 1,25(OH)_2_D_3_ reduces perforin and granzyme B expression, decreases pro-inflammatory cytokine production, thereby attenuating cytotoxic potential (43) (**Figure 1D**). In the B cell compartment, 1,25(OH)_2_D_3_ inhibits maturation and differentiation into plasma cells and memory cells (40) (**Figure 1F**); reduces antibody production by decreasing T follicular helper cell (Tfh) to secrete IL-21 and downregulating C−X−C motif chemokine ligand 13 (CXCL13) expression (44) while promoting the regulatory B cell (Breg) phenotype through inducting IL-10 expression (45). These synergistic effects collectively maintain immune tolerance and regulate autoimmune responses. Recent evidence also reveals that Vitamin D_3_ interacts with the gut microbiome, indirectly influencing adaptive immune function through modulation of microbial composition and metabolite production, adding another layer of complexity to its immunoregulatory repertoire (46). Beyond peripheral immunomodulation, Vitamin D_3_ also contributes to the establishment of central tolerance in the thymus. Thymic epithelial cells express *VDR* and *CYP27B1*, and Artusa et al. demonstrated that *CYP27B1*-deficient mice exhibit impaired differentiation of autoimmune regulator (Aire)-expressing medullary thymic epithelial cells (mTECs), defective negative selection of autoreactive T cells, ultimately leading to autoantibody production and premature thymic aging (47). These findings reveal a potential mechanism by which Vitamin D_3_ prevents autoimmune diseases through regulation of thymic function.

## 4 Regulatory effects of Vitamin D_3_ on key signaling pathways

Vitamin D_3_ influences multiple interconnected signaling pathways that coordinate inflammatory and cellular stress responses. Three pathways — nuclear factor κB (NF-κB), NOD−like receptor protein 3 (NLRP3) inflammasome, and Nuclear Factor Erythroid 2-Related Factor 2 (Nrf2) — are particularly critical in mediating 1,25(OH)_2_D_3_’s immunomodulatory effects:

### 4.1 NF-κB signaling pathway

**Figure 2:**
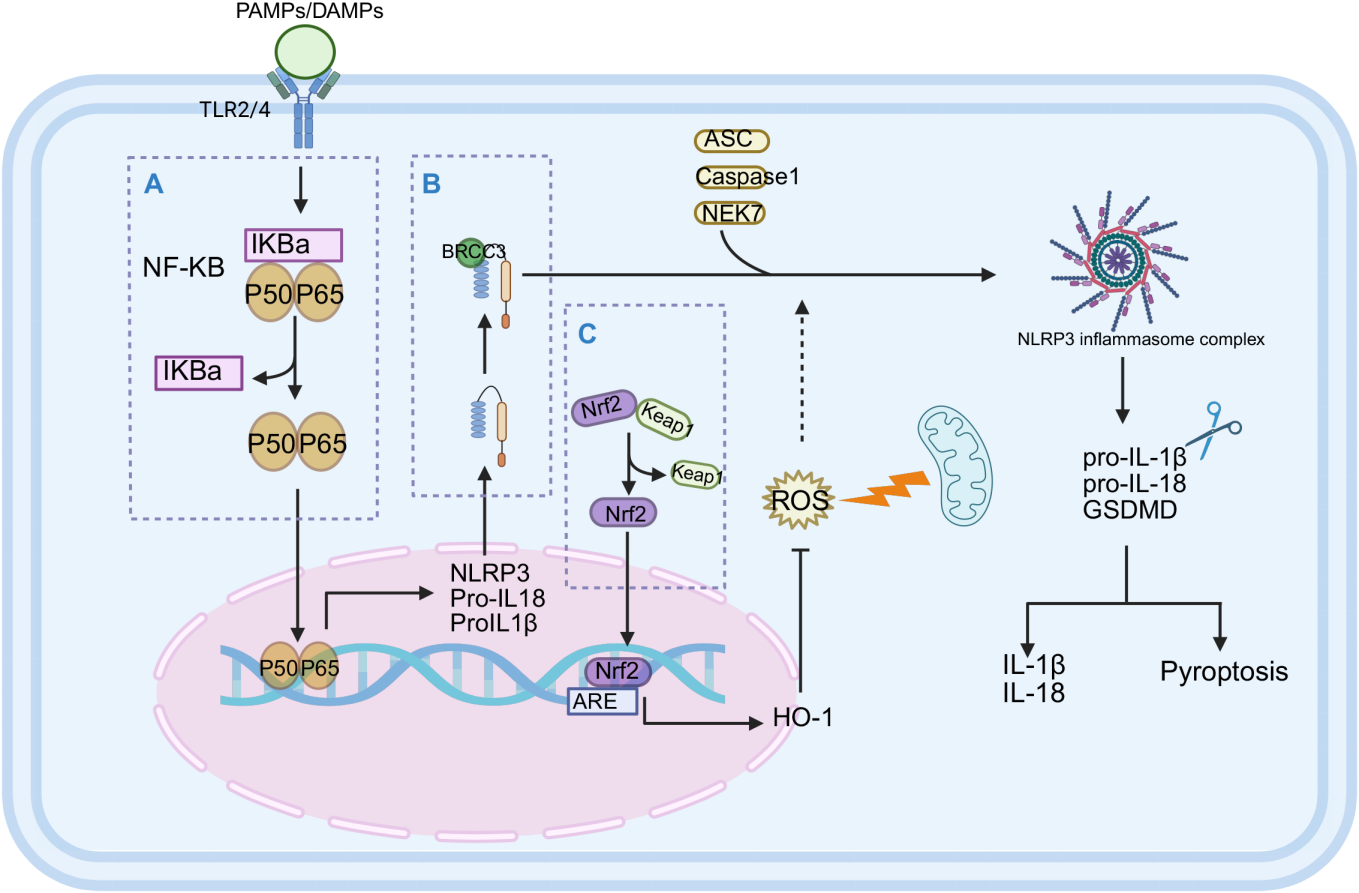
The regulatory mechanism of Vitamin D_3_ on inflammation-related signaling pathways (NF-κB, NLRP3, Nrf2). Vitamin D_3_ mediates multiple anti-inflammatory and antioxidant pathways through VDR:**(A)** NF-κB pathway: Vitamin D_3_/VDR complex up-regulated the expression of IκBα and stabilized its protein, prevented the nuclear translocation of NF-κB subunits (P50, P65), and inhibited the transcription of pro-inflammatory genes (IL-1β, IL-6, TNF-α) (48, 49). **(B)** NLRP3 inflammasome pathway: Vitamin D_3_ inhibits NLRP3 activation through dual mechanisms—blocking BRCC3-mediated NLRP3 deubiquitination to prevent complex formation with ASC and Caspase-1; simultaneously suppressing upstream NF-κB activation signals to reduce NLRP3 inflammasome priming, decreasing IL-1β and IL-18 release (53). **(C)** Nrf2 pathway: Vitamin D_3_ down-regulates the expression of Keap1, a cytoplasmic inhibitor of Nrf2, promotes the nuclear translocation of Nrf2, binds to antioxidant response elements (ARE) (57, 58), activates antioxidant enzymes such as superoxide dismutase (SOD) and heme oxygenase-1 (HO-1), alleviates the damage of oxidative stress to immune cells, and maintains its functional homeostasis (59).

The 1,25(OH)_2_D_3_-VDR complex upregulates Inhibitor of Kappa B Alpha (IκBα) protein levels, stabilizing this inhibitor and preventing nuclear translocation of NF-κB subunits (p50, p65), thereby suppressing transcription of pro-inflammatory genes including IL-1β, IL-6, and TNF-α (48, 49) (**Figure 2A**). Recent studies confirm that 1,25(OH)_2_D_3_ significantly suppresses NF-κB activation in endometrial inflammation (50). Additionally, 1,25(OH)_2_D_3_ has been shown to inhibit microRNA-mediated regulation of the NF-κB signaling pathway, potentially representing a novel mechanism for inflammatory control (51).

### 4.2 NLRP3 inflammasome

Vitamin D_3_ effectively inhibits NLRP3 inflammasome assembly and activation through dual mechanisms: blocking NLRP3 deubiquitination (52) and suppressing upstream NF-κB signaling (53), thereby reducing IL-1β and IL-18 production (**Figure 2B**). Furthermore, Vitamin D_3_ promotes autophagy (54), facilitating clearance of inflammatory triggers, and activates the Hippo−Yes−associated protein 1 (Hippo−YAP1) signaling pathway (55). Recent evidence demonstrates that 1,25(OH)_2_D_3_ enhances the Sirtuin 3 (SIRT3)-Superoxide Dismutase 2 (SOD2) pathway, ameliorating mitochondrial oxidative stress and reducing mitochondrial reactive oxygen species (mtROS) accumulation, thereby indirectly inhibiting NLRP3 inflammasome activation (56).

### 4.3 Nrf2 signaling pathway

VDR activation downregulates Kelch-like ECH-associated protein 1 (Keap1, Nrf2’s cytoplasmic inhibitor), promoting Nrf2 nuclear translocation and activation of antioxidant response elements (57, 58) (**Figure 2C**). This process triggers upregulation of cytoprotective enzymes including superoxide dismutase, catalase, heme oxygenase-1, and glutathione S-transferase (59). In neurodegenerative disease and ischemia models, 1,25(OH)_2_D_3_-mediated Nrf2 activation inhibits ferroptosis, significantly enhancing cell survival rates (60). These antioxidant effects additionally contribute to alleviating chronic inflammation associated with metabolic and cardiovascular diseases (61, 62).

Through coordinated suppression of pro-inflammatory pathways and enhancement of antioxidant defense mechanisms, Vitamin D_3_ establishes a finely tuned balance between immunomodulation and cytoprotection. Its net effect is highly context-dependent, highlighting its unique role as a physiological modulator rather than a simple immunosuppressant. While these molecular mechanisms have been extensively validated *in vitro*, their translation to clinical applications reveals significant complexity requiring careful consideration of individual patient factors.

## 5 Vitamin D_3_ supplementation strategies and influencing factors

Currently, serum 25(OH)D concentration serves as the universally accepted biomarker for assessing Vitamin D_3_ status. According to authoritative recommendations (63, 64) including the 2011 Endocrine Society Clinical Practice Guidelines (ESCPG), 25(OH)D < 20 ng/mL defines deficiency, 20–30 ng/mL indicates insufficiency, and ≥ 30 ng/mL represents sufficiency. However, the 2024 ESCPG no longer endorse specific 25(OH)D thresholds for defining Vitamin D_3_ levels (65), reflecting evolving understanding of Vitamin D_3_ physiology. To maximize health benefits in autoimmune disease, ESCPG recommend maintaining serum 25(OH)D levels at a minimum of 30 ng/mL, with an optimal range of 40–60 ng/mL, while levels up to 100 ng/mL are generally considered safe (63). Multiple international guidelines and expert consensus documents indicate that achieving these target levels typically requires daily supplementation of 1500–2000 international unit (IU) Vitamin D_3_ for general adults (63, 64, 66) (**Box 1**). However, international consensus on optimal supplementation levels remains elusive, with no specific protocols established for different baseline Vitamin D_3_ levels.

### 5.1 Vitamin D_3_ supplementation strategies for various diseases

Several empirical guideline suggests that daily supplementation of 1000 IU Vitamin D_3_ increases serum 25(OH)D levels by approximately 7–10 ng/mL, with greater per-unit dose effects observed at lower baseline levels (63, 67, 68) (**Box 1**). Special populations require individualized approaches: obese individuals need 2–3 times the standard dose to achieve comparable serum concentrations (66, 69, 70) while patients with chronic kidney disease or granulomatous diseases may be particularly susceptible to hypercalcemia, necessitating lower doses or alternative formulations (71) (**Box 1**). For elderly individuals, Gallagher pointed out that daily dosing should not exceed 3000 IU, with serum 25(OH)D levels maintained below 40–45 ng/mL to prevent adverse effects including increased fall risk (72) (**Box 1**). Regarding formulation choices, Vitamin D_3_ is better than Vitamin D_2_ in raising 25(OH)D levels, and multiple studies have demonstrated that daily Vitamin D_3_ supplementation is significantly more efficacious than intermittent high-dose regimens (e.g., weekly or monthly) (73, 74), possibly because the latter fails to maintain stable serum 25(OH)D levels or to achieve benefits on specific endpoints (65, 75). Regular monitoring of serum calcium during supplementation is essential to prevent hypercalcemia from long-term high-dose use. Hypercalcemia is the primary adverse effect of long-term high-dose Vitamin D_3_ supplementation, manifesting as nausea, vomiting, polyuria, polydipsia, and fatigue. Without timely intervention, severe cases may progress to complications including nephrolithiasis, renal insufficiency, cardiac arrhythmias, and soft tissue calcification (76) (**Table 2**). Notably, hypercalciuria may precede hypercalcemia and independently increases the risk of kidney stone formation. High-risk populations primarily include patients with chronic kidney disease, granulomatous diseases (such as sarcoidosis and tuberculosis), and primary hyperparathyroidism. Therefore, regular monitoring of serum and urinary calcium levels during supplementation—particularly when daily doses exceed 4000 IU—is essential (67). If hypercalcemia is detected, supplementation should be discontinued immediately. Ultimately, Vitamin D_3_ should serve as adjunctive therapy in immune-mediated diseases, integrated into comprehensive treatment plans rather than replacing standard treatment. Clinicians should develop individualized dosing strategies based on baseline Vitamin D_3_ levels, genetic polymorphisms, inflammatory markers, season, geographic location, age, BMI, and other relevant factors.

**Table 2:** Comparison of potency, clinical applications, and hypercalcemia risk among various Vitamin D_3_ supplementation forms.

Preparation	Immune/anti-inflammatory	Application direction	Hypercalcemia risk
Calcitriol	High	Psoriasis: Topical application improves skin lesions (3 µg/g, twice daily) (open-label trial) (185)	High: Dose approached threshold (186).
Calcifediol	Moderate	Vitamin D_3_ deficiency (aged ≥11: up to10 μg/day; aged 3–10: up to 5 μg/day) (Scientific Assessment Report) (187); T1DM: children can improve in immune phenotype (100μg/day for 6 months) (RCT) (188)	Lower: High doses or renal impairment still require calcium monitoring.
Cholecalciferol	Moderate	Vitamin D_3_ deficiency(1500–2000 IU/day) (guideline) (63, 70); SLE: Reduces disease activity and improves quality of life (5000 IU/day for 12 weeks) (RCT) (189). T1DM: Improves β-cell and Treg function (3000 IU/day with stable serum 25(OH)D for one year) (RCT) (130). IBD: Improves inflammatory markers in children/adolescents (≥2000 IU/day for 12 weeks) (systematic review) (144); Reduces disease activity and calprotectin in adults (40,000IU/week for 8 weeks)(RCT) (145); Reduces inflammation and relapse rate (mild deficiency: 1000–2000 IU/d; severe deficiency: 2000–4000 IU/d) (expert opinion) (151)	Low: Generally safe at routine supplementation doses.
Calcipotriol	High	Psoriasis: Topical 0.005% (twice daily) significantly improves skin lesions(RCT) (190).	Very low: 1% of calcitriol (191).
Paricalcitol	High	CKD-related secondary hyperparathyroidism (oral 1 μg/day) (guideline) (192)	Lower: one- fourth the dose of calcitriol (193).
Alfacalcidol	Moderate	RA: Mild improvement in clinical indices (1-2 μg/day, 16 weeks) (RCT) (194); T1DM: Improves β-cell function in newly diagnosed children (0.25 μg twice daily) (RCT) (195)	Slightly lower: Hypercalcemia is rare at therapeutic doses.

Calcitriol, 1,25(OH)_2_D_3_; Calcifediol, 25(OH)D_3_; Cholecalciferol, vitamin D_3_.

Box 1 **Clinical Practice Guidelines for Vitamin D_3_ Immunomodulatory Applications**

**Therapeutic Targets:**

Optimal Range: Maintain serum 25(OH)D levels between 40-60 ng/mL for maximal immunomodulatory benefits (guideline) (63).

Minimum Threshold: Ensure levels exceed 30 ng/mL for basic immune function support (guideline) (63).

Safety Ceiling: Avoid exceeding 100 ng/mL to prevent hypercalcemia and adverse effects (guideline) (63).

**Dosing Guidelines:**

General Population: 1500–2000 IU Vitamin D_3_ daily for adults with deficiency or insufficiency (guideline and consensus statement) (63, 64, 66).

Empirical Rule: Each 1000 IU of Vitamin D_3_ daily raises serum 25(OH)D by approximately 7–10 ng/mL (more pronounced at lower baseline levels) (guideline and expert opinion) (63, 67, 68).

Obesity: Obese individuals require 2–3 times higher doses than non-obese individuals to achieve comparable serum 25(OH)D levels (guideline and consensus statement) (66, 69, 70).

Elderly (>75 years): Daily Vitamin D_3_ supplementation should not exceed 3000 IU; maintain serum 25(OH)D below 40–45 ng/mL to minimize fall risk (perspectives) (72).

Chronic Kidney Disease/Granulomatous Disease: Use lower doses or non-calcemic analogues to avoid hypercalcemia (RCT) (71).

**Formulation Choice:** Comparing to Vitamin D_2_, Vitamin D_3_ yields more stable levels, and daily Vitamin D_3_ supplementation is significantly more efficacious than intermittent high-dose regimens (perspective and systematic review) (74, 75)

**Monitoring Requirements:**

25(OH)D Levels: Recheck serum 25(OH)D after 3–4 months of supplementation initiation or dose adjustment (consensus statement) (66).

Calcium Levels: Monitor serum calcium periodically during high-dose supplementation to prevent hypercalcemia.

Disease Markers: Track disease-specific indicators (such as PASI for psoriasis, SLEDAI for SLE, DAS28 for RA, C-peptide/HbA1c for T1DM, PCDAI/PUCAI/calprotectin for IBD) to gauge clinical response.

**Core Clinical Principles:**

Vitamin D_3_ should function as adjunctive therapy integrated into comprehensive disease management strategies, not as monotherapy.

Evidence-based supplementation should target identified deficiency states rather than empirical high-dose administration.

High-risk individuals (e.g., those with obesity, the elderly, or autoimmune diseases) recommends routine Vitamin D_3_ evaluation rather than blind supplementation with routine calcium checks (expert consensus) (66)

Risk-benefit assessment should consider individual patient characteristics, disease severity, and potential drug interactions.

Implementation of biomarker-guided treatment protocols can optimize therapeutic response while minimizing adverse effects.

### 5.2 The effect of gene polymorphism on Vitamin D_3_ supplementation strategy

Genetic studies reveal that polymorphisms in genes including *GC*, *CYP27B1*, *CYP2R1*, *VDR*, *CYP24A1*, *NADSYN1*, *CUBN*, and *DHCR7* significantly influence Vitamin D_3_ status (77), indicating that patient responses to supplementation are genetically determined. Genetic analysis can help determine optimal supplementation doses, enabling early identification and stratified management of “low responders” versus “high responders.” Meta-analyses demonstrate that *TaqI* polymorphism TT variant alleles and *FokI* variant FF alleles correlate positively with Vitamin D_3_ supplementation response, potentially allowing dose reduction (78). Conversely, *FokI* variant CC allele and *ApaI* A allele carriers may require higher maintenance doses (79). Moreover, *DBP* gene variants rs7041 and rs4588 correlate with both 25(OH)D level variation and supplementation effectiveness (80).

### 5.3 Effects of inflammatory markers on Vitamin D_3_ supplementation strategies

Cross-sectional studies consistently show that Vitamin D_3_-sufficient patients (≥30 ng/mL) exhibit significantly lower inflammatory markers including C-reactive protein (CRP) and IL-6 compared to deficient groups (81). A randomized controlled trial demonstrated that patients receiving 2000 IU Vitamin D_3_ daily showed significantly greater IL-6 reduction compared to those receiving 1000 IU daily (82), suggesting higher doses may be necessary for effective systemic inflammation suppression. Evidence supports 20 ng/mL as the minimum target for mechanistic inflammation control, with 28ng/mL as the ideal target for optimizing adaptive immunomodulation (83). Future controlled trials investigating Vitamin D_3_ supplementation efficacy across different baseline levels, inflammatory markers, and immune indicators are essential to determine optimal doses and achieve truly stratified, personalized treatment.

## 6 Clinical translation of Vitamin D_3_

Vitamin D_3_ deficiency is prevalent among patients with immune-mediated diseases. Vitamin D_3_ modulates both innate and adaptive immunity via VDR signaling (Section 3), while suppressing inflammation and enhancing antioxidant defenses through NF-κB, NLRP3, and Nrf2 pathways (Section 4)—mechanisms well-established in experimental studies. However, a substantial translational gap persists: large-scale trials demonstrate highly heterogeneous preventive effects contingent upon baseline Vitamin D_3_ status, *VDR* genetic polymorphisms, disease type, and timing of intervention. This chapter focuses on five diseases—psoriasis, SLE, RA, T1DM and IBD —to address three central questions: how universal immunomodulatory mechanisms translate into therapeutic value; how disease-specific targets influence efficacy profiles; and how biomarkers can enable precision therapy. Despite their diverse clinical manifestations, the shared underlying immune dysregulation provides a rationale for cross-disease application of Vitamin D_3_. Therapeutic variability depends on disease-specific targets, including keratinocytes in psoriasis, pancreatic β-cells in T1DM and intestinal barrier integrity in IBD. Following this framework, we systematically discuss the disease-specific mechanisms, genetic backgrounds, clinical evidence, and stratified therapeutic strategies for each condition.

### 6.1 Psoriasis

**Figure 3:**
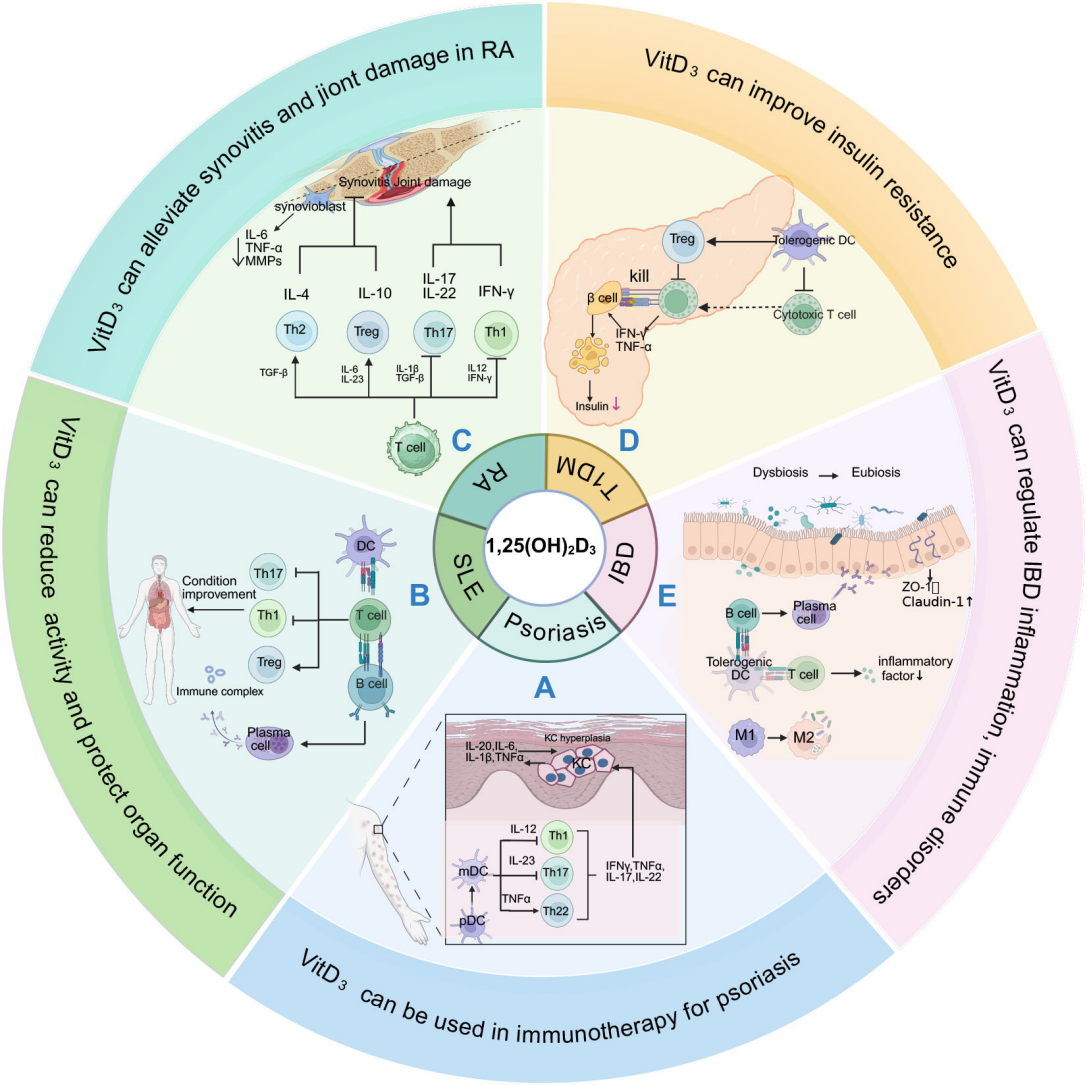
Immunoregulatory targets and pathways of Vitamin D_3_ in major diseases. Vitamin D_3_ regulates disease progression through multiple targets: **(A)** Psoriasis: Vitamin D_3_ inhibits the abnormal proliferation of keratinocytes (87), reduces the release of inflammatory factors such as IL-20 and IL-6, and also inhibits the maturation and antigen presentation of dendritic cells (90), promotes the shift of Treg/Th17 balance to immune tolerance (89), and reduces the stimulation of inflammatory mediators (IFN-γ, TNF-α, etc.) on keratinocytes. **(B)** SLE: inhibit the differentiation of CD4 ^+^ T cells into Th1/Th17 and promote the expansion of Treg (99), while inhibiting B cell activation (100) and autoantibody (such as anti-dsDNA antibody) production (101); **(C)** RA: Vitamin D_3_ regulates Th17/Treg balance, reduces pro-inflammatory cytokines (IL-17, IFN-γ, etc.), increases anti-inflammatory cytokines (IL-4, IL-10, etc.), and reduces synovial fibroblasts. The production of IL-6, TNF-α and matrix metalloproteinase (MMP) jointly reduce synovitis and joint destruction (108). **(D)** T1DM: Vitamin D_3_ activates β-cell VDR to enhance insulin secretion, activates pPPAR-δ to improve tissue insulin sensitivity (121), inhibits killer T cells to reduce β-cell damage, inhibits pathogenic lymphocyte proliferation and pro-inflammatory cytokines (IFN-γ, TNF-α) release, and induces immune tolerance (122); **(E)** IBD: Vitamin D_3_ regulates intestinal innate immunity by upregulating NOD2 expression and activating the NF-κB pathway (30); induces tight junction protein (ZO-1, claudin-1) expression to enhance intestinal epithelial barrier integrity (142); suppresses pro-inflammatory cytokine (TNF-α, IL-1β, IL-6) production; modulates gut microbiota composition to promote beneficial bacterial colonization, collectively maintaining intestinal immune homeostasis (143).

Vitamin D_3_ deficiency is prevalent among psoriasis patients and may correlate with disease activity. Cross-sectional studies show that approximately 57.8% of psoriasis patients have Vitamin D_3_ deficiency, rising to 80.9% during winter months with minimal sunlight, significantly exceeding healthy population rates (84). Genetic studies further reveal individual differences in Vitamin D_3_ metabolism: *VDR* gene *ApaI* and *TaqI* polymorphisms not only correlate with psoriasis susceptibility but also affect patient 25(OH)D levels, with *ApaI* AA genotype patients showing the lowest serum 25(OH)D levels (85). Notably, disease severity may also influence Vitamin D_3_ levels, with observations showing greater 25(OH)D level increases in moderate psoriasis patients compared to mild and severe cases (86). Mechanistically, Vitamin D_3_ acts through multiple pathways in psoriasis (**Figure 3A****) (****Table 3**): regulating keratinocyte growth and differentiation (87), enhancing antimicrobial barrier function through LL-37 induction (88), suppressing pathogenic Th1/Th17 responses while promoting Tregs (89), and inhibiting DCs and macrophage activation (90). These mechanisms collectively address psoriasis-specific epidermal and immunological abnormalities.

**Table 3:** Summary of clinical application evidence of Vitamin D_3_ in different diseases.

Disease type	Main mechanism	Summary of clinical evidence
Psoriasis	Regulate the differentiation of keratinocytes (87); induction of LL-37 enhances the barrier (88); inhibition of Th1/Th17; promote Treg (89); inhibit DC/macrophage activation (90).	Local medication [1,25(OH)_2_D_3_ analogues] has a clear effect, and combined hormone or phototherapy is the standard regimen (91, 92); Oral supplements have a weak effect and are only used as an aid.
SLE	Promote Treg; inhibition of Th17 and Tfh cells (99); reduce B cell autoantibodies (100); regulate apoptosis pathway (101); reversal of abnormal DNA methylation in T cells (102).	Observational studies have shown a negative correlation. The results of the supplementary test were contradictory, and some showed slight improvement in SLEDAI score and anti-dsDNA antibody (103).
RA	Inhibit Th17; promoting Treg (107); reduce IL-6, TNF-α and other inflammatory mediators; regulating RANKL/OPG balance (108).	There are differences in evidence. Some studies have shown that high doses can improve symptoms; But individual differences are significant (113, 114).
T1DM	Enhance insulin secretion via calcium signaling (120); improve insulin sensitivity (activate PPAR-δ) (121); reduce islet inflammation (122).	Early supplementation in newly diagnosed patients helps preserve residual β-cell function (132);Effects on glycemic control in established T1DM remain controversial.
IBD	Activate NOD2-NF-κB pathway (30); induce tight junction proteins for barrier integrity (142); modulate gut microbiota (143).	Vitamin D_3_ sufficiency is associated with reduced IBD risk (especially UC), supplementation elevates serum 25(OH)D and alleviates inflammation (139, 141, 146).

Topical 1,25(OH)_2_D_3_ analogues (such as calcipotriol) are first-line treatments for psoriasis, often used in combination with corticosteroids (91) or phototherapy (92) to enhance efficacy. Studies show calcipotriol monotherapy can achieve 59% reduction in Psoriasis Area and Severity Index (PASI) after 8 weeks of treatment. One trial demonstrated that a 4-week course of calcipotriol–betamethasone combination therapy led to Physician Global Assessment (PGA) 0/1 (clear or nearly clear) rates of 72.6%, 56.5%, and 66.7% in mild, moderate, and severe psoriasis patients, respectively (93). Notably, topical medication efficacy is primarily driven by high concentrations achieved at lesion sites, with minimal correlation to patient serum 25(OH)D levels.

According to current international S3 guidelines and American Academy of Dermatology guidelines (94) for mild, localized patients: 1,25(OH)_2_D_3_ analogue monotherapy is preferred, applied twice daily. For moderate patients: 1,25(OH)_2_D_3_ analogues combined with topical corticosteroids (such as combination preparations) are recommended to enhance efficacy, with a transition to intermittent use after initial response to maintain efficacy and reduce side effects. For severe patients, systemic therapy should be the primary approach, with topical combination preparations as adjunctive treatment. This further confirms that Vitamin D_3_ should serve as adjunctive therapy rather than primary treatment, mainly correcting deficiency. Extensive clinical trials support the safety and efficacy of topical 1,25(OH)_2_D_3_ analogues, whereas evidence for benefit from oral supplementation is primarily observational (95). Recent studies also indicate that Vitamin D_3_ status can affect response to biologic therapies, suggesting that maintaining adequate 25(OH)D may optimize outcomes when using TNF-α or IL-17 inhibitors (96).

### 6.2 Systemic lupus erythematosus

Vitamin D_3_ deficiency is extremely prevalent in SLE patients. Studies indicate that up to 96% of SLE patients have Vitamin D_3_ insufficiency (25(OH)D < 30 ng/mL), with 27% showing severe deficiency (25(OH)D < 15 ng/mL) (97). Observational studies consistently find negative correlations between serum 25(OH)D levels and disease activity in SLE patients (98). The immunomodulatory mechanisms of Vitamin D_3_ align highly with SLE pathophysiology (**Figure 3B****) (****Table 3**): promoting Treg cells development while suppressing Th17 and Tfh (99); reducing B cell autoantibody production (100); regulating apoptotic pathways by upregulating anti-apoptotic protein B-cell lymphoma 2 (Bcl-2) and downregulating pro-apoptotic mediators (101); and reversing lupus T cell-specific DNA methylation abnormalities (102). These effects collectively reduce autoimmune response activity and mitigate inflammatory tissue damage.

However, clinical research on Vitamin D_3_ supplementation yields inconsistent results. Vitamin D_3_ supplementation as adjunctive therapy may modestly reduce Systemic Lupus Erythematosus Disease Activity Index (SLEDAI) scores and improve fatigue symptoms but cannot replace standard immunosuppressive regimens (103) (**Box 1**). Different studies show contradictory effects on anti-dsDNA antibodies and complement component 4, with overall conclusions showing no significant improvement. Some studies also failed to demonstrate that Vitamin D_3_ supplementation improves various biomarkers in SLE patients; This suggests that its benefits may be limited to specific subgroups (such as severely deficient patients or those with seasonal variation).

Currently, no consensus guidelines exist for immunomodulatory dosing of Vitamin D_3_ in autoimmune rheumatic diseases, despite *in vitro* experiments suggesting high-dose Vitamin D_3_ may induce immunomodulatory effects. Research indicates that Vitamin D_3_ supplementation shows better therapeutic effects in patients with lower Vitamin D_3_ levels (104). For children, especially those on long-term corticosteroids, Vitamin D_3_ requirements are at least twice the age-recommended intake (approximately 2000 IU/day) (63). Additionally, certain polymorphisms (such as *VDR BsmI* and *FokI* variants) may influence SLE risk in Asian populations (105), and factors such as *VDR* gene polymorphisms can predict treatment response. Therefore, future trials should stratify subjects based on baseline Vitamin D_3_ levels, gene polymorphisms, and concomitant medications to identify populations with maximal benefit.

### 6.3 Rheumatoid arthritis

Vitamin D_3_ deficiency is common in RA patients. Observational studies suggest associations between Vitamin D_3_ status and RA risk. For example, the large-scale Iowa Women’s Health Study found that higher dietary Vitamin D_3_ intake correlated with 34% reduced RA risk (106). Vitamin D_3_ supplementation may provide anti-inflammatory and bone-protective effects. Mechanistic studies show (**Figure 3C****) (****Table 3**) that Vitamin D_3_ suppresses Th17 differentiation and matrix metalloproteinase expression while promoting Treg responses (107), and maintains bone health through regulation of Receptor Activator of Nuclear Factor Kappa-B Ligand (RANKL)/osteoprotegerin (OPG) (108).

However, clinical studies on Vitamin D_3_ treatment in established RA have yielded inconsistent conclusions. One randomized controlled trial found no significant effects of Vitamin D_3_ supplementation on Erythrocyte Sedimentation Rate (ESR) and Disease Activity Score in 28 Joints (DAS28) (109, 110). This may relate to differences in study design, dosing, duration, and patient baseline characteristics. Evidence for optimal dosing remains insufficient, and gene polymorphisms in Vitamin D_3_ metabolic pathways may influence effects (111). A national randomized controlled trial (Vitamin D_3_ and Omega-3 Trial) including over 25,000 participants showed that daily supplementation with 2000 IU Vitamin D_3_, over a median follow-up of 5.3 years, significantly reduced overall autoimmune disease incidence (including RA) by 22% (7). In another randomized double-blind placebo-controlled study, adding a single 300,000 IU Vitamin D_3_ dose to standard therapy improved patient overall health status (mean serum 25(OH)D levels increased from baseline 16 ± 4 ng/mL to endpoint 28 ± 4.3 ng/mL), with no adverse reactions over three months (112). Dose-response studies show optimal anti-inflammatory effects with daily supplementation below 3,500 IU, with diminished effects above this dose (113); another study suggests better efficacy with weekly supplementation below 50,000 IU (114). Clinical practice should include regular Vitamin D_3_ level monitoring with dose adjustments based on individual circumstances to maintain serum 25(OH)D within appropriate ranges without inducing hypercalcemia. Given these complex dose-response relationships and individual differences, implementing stratified Vitamin D_3_ treatment strategies for RA patients is crucial.

### 6.4 Type 1 diabetes mellitus

Type 1 diabetes mellitus (T1DM) is a chronic autoimmune disorder characterized by pancreatic β-cell destruction and absolute insulin deficiency, predominantly affecting children and adolescents (115). Global T1DM prevalence reached approximately 8.4 million in 2021, with projections estimating 13.5–17.4 million cases by 2040 (116).

Vitamin D_3_ deficiency is highly prevalent among T1DM patients, with meta-analysis indicating approximately 45% of children/adolescents with T1DM exhibit Vitamin D_3_ insufficiency (117). Observational studies consistently demonstrate an inverse association between serum Vitamin D_3_ levels and T1DM risk (118). Meta-analyses indicate that Vitamin D_3_ supplementation during infancy may reduce T1DM risk by approximately 30% (119). These epidemiological findings suggest a potentially significant role for Vitamin D_3_ in T1DM prevention. The protective effects of Vitamin D_3_ in T1DM involve multiple mechanisms (**Figure 3D**) (**Table 3**). First, 1,25(OH)_2_D_3_ enhances β-cell insulin secretion through calcium signaling regulation and insulin gene transcription (120). Second, 1,25(OH)_2_D_3_ improves peripheral insulin sensitivity via Peroxisome Proliferator−Activated Receptor δ (PPAR-δ) activation (121). Third, 1,25(OH)_2_D_3_ protects pancreatic β-cells from autoimmune destruction by reducing pro-inflammatory cytokines (TNF-α, IL-1β) (122) and exerting antioxidant effects (123). Additionally, 1,25(OH)_2_D_3_ downregulates antigen-presenting molecules such as cathepsin G, thereby suppressing autoreactive T-cell activation and delaying β-cell immune destruction (124). The Vitamin D_3_ regulation of thymic central tolerance described above (Section 3.2) is directly relevant to T1DM prevention: Artusa et al. also found that aged *CYP27B1*-knockout mice developed anti-islet autoantibodies and glucose intolerance (47). Since infancy represents the period of peak thymic activity, Vitamin D_3_ supplementation during this window helps maintain normal negative selection, preventing autoreactive T cells from escaping to attack pancreatic β-cells. These mechanisms are particularly critical in early disease stages when 60–95% of β-cells are already compromised at diagnosis (125), as Vitamin D_3_ supplementation may preserve residual β-cell function and exert immunomodulatory effects.

Numerous reviews and meta-analyses have established associations between genetic variants in Vitamin D_3_-related genes and diabetes susceptibility. Studies have shown that among children with T1DM in China, carriers of the C allele of *CYP2R1* (rs1993116) have a higher risk of developing T1DM than those carrying the T allele (126). A prospective cohort study of 101 newly diagnosed T1DM children demonstrated that adequate Vitamin D_3_ status (≥30 ng/mL) and VDR gene *FokI* and *TaqI* polymorphisms were associated with better preservation of residual β-cell mass and function (127). However, findings remain inconsistent. For instance, a large Mendelian randomization study found no significant association between these polymorphisms and T1DM risk, suggesting that the role of genetic background requires further elucidation (128).

Regarding clinical interventions, the efficacy of Vitamin D_3_ supplementation on glycemic control in established T1DM remains controversial. Some studies indicate that Vitamin D_3_ supplementation (cholecalciferol 2000 IU/day) elevates serum 25(OH)D levels without significantly improving glycemic parameters such as Hemoglobin A1c (HbA1c) (129). Conversely, other studies report reductions in fasting glucose, mean daily glucose, and insulin requirements (130), along with attenuated inflammatory responses during the honeymoon period (the early post-diagnosis phase characterized by residual β-cell function and lower insulin requirements) through decreased serum TNF-α levels, thereby prolonging honeymoon duration (131). Importantly, the protective effects of Vitamin D_3_ on pancreatic β-cell function are evident only within the first year of disease onset (newly diagnosed T1DM patients) (132), and 25(OH)D levels directly correlating with fasting C-peptide levels in newly diagnosed adolescents and adults (133). Additionally, accumulating evidence suggests that combined therapy with Vitamin D_3_ and dipeptidyl peptidase-4 inhibitors (DPP-4i) may preserve β-cell function in adults with latent autoimmune diabetes (134). Major diabetes associations have not yet recommended routine Vitamin D_3_ supplementation for improving glycemic control in T1DM, primarily due to evidence heterogeneity and undefined optimal dosing (129, 135). Nevertheless, low Vitamin D_3_ status may be associated with increased diabetic ketoacidosis risk (136), hypoglycemic events, and poor metabolic control (137)in newly diagnosed children with T1DM. Therefore, correcting Vitamin D_3_ deficiency remains important for overall disease management and patient health, although optimal supplementation dosages and intervention timing require validation through larger prospective studies.

### 6.5 Inflammatory bowel disease

Inflammatory bowel disease (IBD) encompasses chronic relapsing inflammatory disorders of the gastrointestinal tract, affecting approximately 4.9 million individuals worldwide, primarily comprising Crohn’s disease (CD) and ulcerative colitis (UC) (138). Animal models and epidemiological studies consistently demonstrate significant associations between low Vitamin D_3_ levels and IBD risk (139). An Italian study reported mean Vitamin D_3_ concentrations of 18.9 ± 10.2 ng/mL in IBD patients, significantly lower than healthy controls (140). Prospective studies indicate that each 1-μg increment in Vitamin D_3_ intake reduces IBD risk by 51%, with this association persisting after adjustment for confounding factors (141). Vitamin D_3_ participates in IBD pathophysiology through multiple mechanisms (**Figure 3E****) (****Table 3**). First, 1,25(OH)_2_D_3_ exerts immunomodulatory effects by enhancing antimicrobial protein expression and activating the nucleotide-binding oligomerization domain containing 2 (NOD2)-HBD2 signaling pathway (30): 1,25(OH)_2_D_3_ induces NOD2 expression, which activates NF-κB signaling to induce HBD2 production (30). This synergistic enhancement is abolished in CD patients with homozygous NOD2 mutations (29), providing a molecular link between Vitamin D_3_ deficiency and CD genetic susceptibility, thereby regulating immune cells and inflammatory cascades (30). Second, 1,25(OH)_2_D_3_ strengthens intestinal epithelial barrier function by inducing tight junction protein expression, including zonula occludens-1 (ZO-1) and claudin-1 (142). Additionally, Vitamin D_3_ may further promote intestinal health through gut microbiota modulation (143). Notably, these effects may exhibit individual variability influenced by factors including sex, hypertension, and smoking.

Clinical efficacy studies of Vitamin D_3_ supplementation demonstrate considerable heterogeneity. In pediatric and adolescent populations, meta-analyses indicate that Vitamin D_3_ supplementation (≥2000 IU/day for 12 weeks) significantly improves serum 25(OH)D concentrations and inflammatory markers with favorable safety profiles (144). In adults, supplementation with 40,000 IU/week for 8 weeks reduces disease activity indices, calprotectin, and serum C-reactive protein (CRP) levels while increasing albumin concentrations (145). Cohort studies reveal that elevated 25(OH)D levels correlate with reduced bowel resection risk, decreasing overall IBD risk by 34% and UC risk by 46%, although this association is not significant in CD patients (146). However, some studies found no significant correlations between serum 25(OH)D changes and endoscopic findings, calprotectin, Pediatric Crohn’s Disease Activity Index (PCDAI), or Pediatric Ulcerative Colitis Activity Index (PUCAI) scores, highlighting the need for further research to clarify clinical utility (147). *VDR* gene polymorphisms are closely associated with IBD susceptibility. Meta-analyses demonstrate that the *FokI* polymorphism ff allele confers elevated UC risk in Asian populations, while the *ApaI* polymorphism a allele confers CD protection in European populations (148). The *TaqI* polymorphism TT genotype associates with increased UC and CD risk in males, while the *BsmI* B allele correlates with elevated CD risk in East Asian populations (149). These genetic findings further support the critical role of Vitamin D_3_ in IBD pathogenesis.

Despite accumulating evidence, while the American Gastroenterological Association recommends Vitamin D_3_ monitoring for all IBD patients, optimal supplementation dosages remain unestablished (150). Ananthakrishnan proposes that individualized dosing based on baseline Vitamin D_3_ status: 1000–2000 IU/day for mild deficiency, 2000–4000 IU/day for severe deficiency until target achievement, followed by 1000 IU/day maintenance (151). Given the substantial individual variability in Vitamin D_3_’s role in IBD prevention and management, high-quality randomized controlled trials are needed to define stratified management strategies, including optimal dosing, treatment timing, and subtype-specific approaches for different IBD phenotypes.

Synthesizing evidence across five diseases, Vitamin D_3_ clinical translation exhibits a characteristic coexistence of “mechanistic certainty” and “therapeutic heterogeneity.” Topical analogs demonstrate robust efficacy in psoriasis, whereas oral supplementation in SLE, RA, T1DM, and IBD is highly dependent on baseline Vitamin D_3_ status, VDR polymorphisms, and disease stage. This reveals a core therapeutic logic: efficacy is pronounced with topical administration targeting defined cellular populations, while systemic immunomodulation requires precise identification of responder subgroups. The clinical translational significance of Vitamin D_3_ lies in its role as a critical nexus linking nutrition, immunity, and metabolism, offering a low-cost, low-toxicity adjunctive therapeutic option for immune-mediated diseases. Current guidelines generally do not recommend routine Vitamin D_3_ status assessment in the general asymptomatic population, given the high testing costs and absence of universally accepted diagnostic thresholds (152–154). While routine screening for high-risk groups (including individuals with obesity, elderly patients, those on chronic glucocorticoid therapy, and patients with autoimmune or chronic diseases) remains controversial, the latest international expert consensus advocates baseline 25(OH)D evaluation with individualized supplementation protocols tailored to baseline levels, body weight, concurrent medications, and clinical context (66). The approach of empirical supplementation coupled solely with serum calcium monitoring is not recommended, as hypercalcemia represents a late manifestation of Vitamin D_3_ intoxication and does not reflect Vitamin D_3_ status or tissue-level activity (68, 155). More importantly, this therapeutic heterogeneity has catalyzed a paradigm shift from empirical supplementation toward precision medicine—enabling individualized stratified treatment through integration of biomarkers including baseline 25(OH)D levels, VDR genotypes, and disease activity indices. This approach not only optimizes the clinical application of Vitamin D_3_ itself but also establishes a methodological framework for translational research on other nutrients and immunomodulatory agents. Future research should focus on developing multidimensional predictive models, defining disease-specific dosing regimens, and elucidating synergistic mechanisms with biologics, ultimately achieving the transition from “one-size-fits-all” to precision intervention—thereby opening new avenues for the comprehensive management of immune-mediated diseases.

## 7 Novel delivery systems

Traditional oral Vitamin D_3_ preparations exhibit an absorption efficiency of approximately 50% (156), with substantial inter-individual variability (coefficient of variation ~47%) (157). Multiple factors contribute to this absorption variability: dietary fat intake (co-administration with fat-containing meals increases serum 25(OH)D levels by 32–50% (158, 159)), gastrointestinal functional status (including bile salt availability, intestinal transit time, and mucosal integrity), genetic polymorphisms in Vitamin D_3_ metabolic genes (*CYP2R1*, *CYP27B1*, *VDR*, and *GC*), and patient-specific factors (obesity, advanced age, and hepatic or renal dysfunction) (1, 160).Furthermore, the intrinsic physicochemical properties of Vitamin D_3_—high hydrophobicity, photochemical instability, and hepatic first-pass metabolism—further limit absorption efficiency in conventional formulations. To address these challenges, several novel delivery systems have been developed in recent years, including nanoemulsions, nanostructured lipid carriers (NLCs), liposomes, intranasal sprays, and twin-screw extrusion formulations (**Table 4**). These systems significantly enhance the dissolution and transmembrane transport efficiency of Vitamin D_3_ in the gastrointestinal tract through various mechanisms: reducing particle size, increasing surface area, optimizing mucosal transport, or bypassing first-pass metabolism, thereby improving its pharmacokinetic properties.

**Table 4:** Comparison of new delivery systems for Vitamin D_3_.

Type	Properties	Optimization	Pharmacokinetic	Application
NE	Oil−phase nano−droplets (20–200 nm) require surfactants for stability (161).	Ultra−fine, stable droplets, boost surface area, improve dissolution and absorption.	Cmax increase 43%, AUC increase 36% (175), higher serum levels (162), dose halved (176).	Oral liquid drop dosage form, improve the bioavailability (162, 163).
NLC	Solid/liquid lipid NLC (120–200 nm) with long-chain fatty acids and emulsifiers (177).	Bypasses first−pass, extends intestinal retention, evades RES clearance, and blocks P−gp efflux (178).	Faster onset; prolonged exposure; improved long-term stability (179).	Oral capsule/suspension improve exposure and immune regulation efficacy (163).
TSE-SD	Co-extruded with a polymer matrix via low-temperature processing to prevent degradation (168).	Uniform drug molecule distribution speeds dissolution; the formulation is stable, compressible, and easily scalable.	Disintegration within 1 min; >75% release within 15 min; bioavailability comparable to solution (176, 180).	Oral solid preparations improve stability, suitable for industrial production.
IN-Lipo	Phospholipid bilayer nanovesicles can encapsulate Vitamin D_3_.	Extend nasal mucosal residence, promote epithelial transport, induce mucosal immune tolerance (167).	~4-fold increase in AUC (16); faster absorption; higher plasma concentration (181).	Allergic rhinitis and mucosal inflammation boosts efficacy, acts fast (182).
IN-NS	Aqueous solution or microemulsion Form micro spray particles by the atomizer.	Bypasses first−pass, absorb rapidly; mucosal delivery quickly raise blood concentration.	Significantly elevated Cmax; Faster onset (183).	As a Vitamin D_3_ adjunct, peak plasma level is about 1.7 times that of oral dosing.
NE	Attach a fluorescent group molecule to retain the binding activity of VDR.	Visualization of *in vivo* distribution, trace pharmacokinetics (184).	Enables real-time visualization of pharmacokinetic profiles.	Analyze the role of Vitamin D_3_ in immune organs and screen analogues.

NE, nanoemulsion; NLC, nanostructured lipid carrier; TSE-SD, twin-screw extrusion solid dispersion; IN, intranasal; Lipo, liposome; NS, nasal spray; Vitamin D_3_-FL, Vitamin D_3_–fluorescein probe; PK, pharmacokinetics; Cmax, peak plasma concentration; AUC, area under the curve.

**Figure 4:**
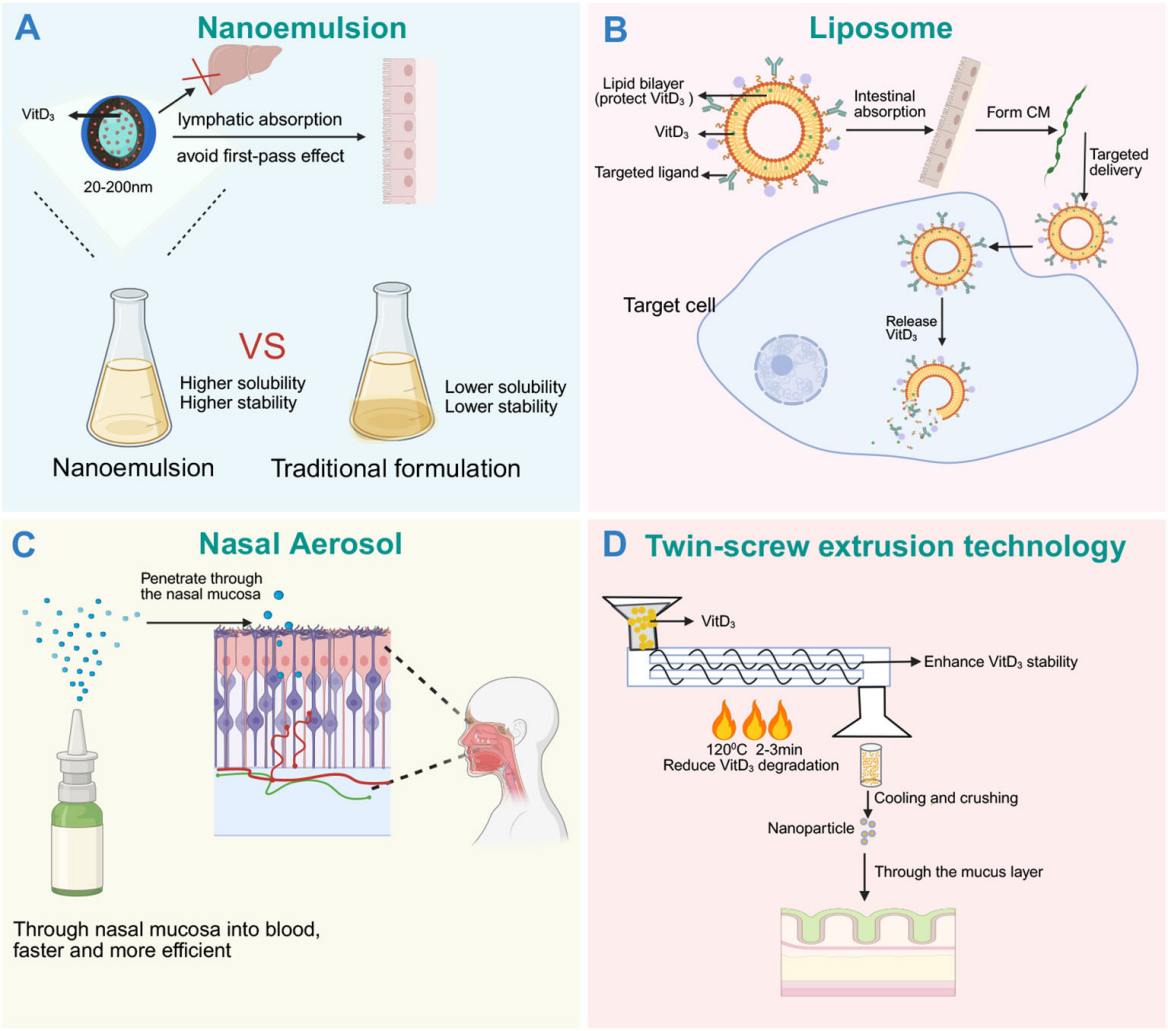
Schematic diagram of structure and mechanism of Vitamin D_3_ novel drug delivery system. **(A)** The nanoemulsion encapsulates Vitamin D_3_ with 20–200 nm lipid nanodroplets, enhances intestinal penetration with surfactants, and bypasses the first-pass effect of the liver through lymphatic transport (161); **(B)** the liposome uses the phospholipid bilayer structure to protect Vitamin D_3_ from degradation, which can be combined with the targeted ligand to achieve accurate delivery; **(C)** Nasal spray Vitamin D_3_ was made into atomized particles by nasal spray, so that it can quickly enter the blood through the rich capillaries of the nasal mucosa to avoid the first-pass effect. Local high concentration of Vitamin D_3_ can activate the VDR of immune cells in the local area and enhance mucosal immune tolerance and barrier function (167); **(D)** Twin-screw extrusion technology was used to prepare nanoparticles by blending Vitamin D_3_ with polymer carriers under low temperature conditions of 120 °C and 2-3min, which not only reduced Vitamin D_3_ degradation, enhanced stability, but also improved cell penetration ability (168, 169). Compared with traditional formulations with low solubility and poor stability, the new delivery system significantly optimizes the absorption and utilization of Vitamin D_3_ through the above mechanism.

Nanoemulsions and nanostructured lipid carriers (NLCs) encapsulate Vitamin D_3_ in nanodroplets (20–200 nanometers), promoting intestinal lymphatic transport and bypassing hepatic first-pass metabolism (161). These delivery systems not only increase surface area but also improve dissolution and absorption (**Figure 4A**). Studies show that nanoemulsion - based delivery systems enhance *in vitro* bioavailability by 3.94-fold (p<0.05) (162), with animal experiments showing approximately doubled serum 25(OH)D levels compared to crude emulsions (162). Human trials demonstrate that micellar Vitamin D_3_ increases serum 25(OH)D levels nearly 1.6-fold compared to traditional fat-soluble Vitamin D_3_ (163). Another animal experiment confirmed that compared to conventional Vitamin D_3_ preparations, oral NLCs administration significantly increased Vitamin D_3_ plasma peak concentration and total exposure, thereby enhancing immunomodulatory effects (164).

Liposomal vesicles enable targeted delivery and avoid rapid clearance, while intranasal sprays allow rapid absorption through nasal mucosa (**Figures 4B, C**), particularly suitable for therapeutic scenarios requiring rapid supplementation (165). Using specialized atomization spray devices (particle size ~0.7 μm) combined with penetration enhancers or mucoadhesive agents, Vitamin D_3_ absorption efficiency can be further improved (166). Beyond systemic effects, intranasal Vitamin D_3_ also produces local immunomodulatory effects — enhancing nasal mucosal antimicrobial peptide secretion while reducing pro-inflammatory mediator production (167).

Twin-screw extrusion technology can produce solid dispersions of Vitamin D_3_ with polymers at low temperatures, avoiding degradation while yielding stable tablets and capsules suitable for large-scale production (168) (**Figure 4D**). By uniformly dispersing molecules within polymer matrices, both crystal size reduction and solubility enhancement are achieved (169). Through optimization of feed liquid/solid ratio and the addition of excipients, Vitamin D_3_ stability and *in vitro* release performance can be further improved (168). In one study using this technology to prepare Vitamin D_3_-iron composite particles, Vitamin D_3_ retention reached 99.8% (170).

These innovative delivery technologies not only promise improved absorption and reliability but also enable tissue-targeted delivery and rapid onset, paving the way for personalized Vitamin D_3_ therapy. Future research directions include conjugating Vitamin D_3_ derivatives with antibodies or peptides for cell-specific targeting, and developing stimulus-responsive systems that release Vitamin D_3_ based on pH or redox changes in diseased tissues.

## 8 Challenges and issues

Despite abundant mechanistic research and encouraging preclinical data, the widespread clinical application of Vitamin D_3_ as an immunotherapeutic agent confronts multiple formidable challenges (**Table 5**). Although Vitamin D_3_ supplementation at recommended doses generally maintains a favorable safety profile, but hypercalcemia risk remains, especially when serum 25(OH)D concentrations exceed 150 ng/mL (171). However, in high-risk populations—including patients with chronic kidney disease, granulomatous diseases, and primary hyperparathyroidism—hypercalcemia may develop at substantially lower thresholds due to impaired Vitamin D_3_ metabolism or unregulated extrarenal 1α-hydroxylase activity. Notably, clinically used 1,25(OH)_2_D_3_ analogues (distinct from Vitamin D_3_ supplementation) typically exhibit comparable or greater immunomodulatory potency with reduced calcemic activity, thereby providing a wider therapeutic window (**Table 2**). Consequently, rigorous monitoring of serum 25(OH)D concentrations and careful dose titration are imperative to ensure safety.

**Table 5:** Challenges and future research directions of clinical application of Vitamin D_3_ immunomodulation.

Challenge	Description	Suggested research direction
Limited use	Long-term or high-dose will elevates the risk of hypercalcemia, particularly in high-risk populations.	Development of selective VDR modulators (analogues) to achieve immune activation and separation of blood calcium effects.
Individual differences are significant.	Genetic polymorphisms (VDR, CYP27B1, DBP) lead to a dose-response difference of up to 6 times; it was affected by baseline level, age, BMI and complications.	Using precision medicine: Combining genotyping, pharmacogenomics analysis, and machine learning algorithms to predict responders and develop individualized dosing regimens.
Contradictions in clinical results	The effect of *in vitro*/animal model is difficult to transform in complex human environment. There is a redundant compensation mechanism in the disease (172); many large RCTs have negative results in unscreened populations.	Carry out large-scale, well-designed RCTs for people with Vitamin D_3_ deficiency; pay attention to the endpoint indicators with clinical significance; explore combined treatment strategies with standard therapies (such as methotrexate, glucocorticoids).
Limitations of preparations	The bioavailability of traditional oral preparations is low and unstable. Rely on complete gastrointestinal absorption function.	To promote and apply new delivery systems to improve bioavailability, stability and medication compliance.
The mechanism of action is complex	Vitamin D_3_ has pleiotropic and context-dependent regulation of the immune system, and its net effect is difficult to predict in different disease stages and environments.	Strengthen basic mechanism research, clarify tissue and cell-specific reactions; explore applications in new areas (such as post-viral syndrome, immunosenescence).

Individual responses to supplementation exhibit remarkable heterogeneity. Genetic polymorphisms in *CYP27B1* and *VDR*, combined with patient-specific factors including baseline Vitamin D_3_ status, age, adiposity, and comorbidities, collectively drive substantial inter-individual variation in treatment response (79). Despite this well-documented variability, current clinical practice rarely incorporates pharmacogenetic testing into dose optimization protocols, representing a significant missed opportunity for personalized therapy.

The pleiotropic effects of Vitamin D_3_ across multiple interconnected immune pathways render overall therapeutic efficacy difficult to predict. Promising effects observed in controlled *in vitro* systems or animal models often fail to translate into meaningful clinical benefits, due largely to redundant compensatory mechanisms in human disease pathophysiology (172). This translational gap is reflected in conflicting trial results, where mechanistic sub-studies demonstrate clear immunological effects that do not always correspond to tangible clinical benefits.

Furthermore, conventional Vitamin D_3_ formulations suffer from inherent pharmaceutical limitations, including poor stability and dependence on intact gastrointestinal absorption mechanisms — systems that are frequently compromised in target patient populations. Multiple large-scale clinical trials have demonstrated that Vitamin D_3_ supplementation shows no significant effects on diabetes incidence, cardiovascular events, or autoimmune disease prevention in unselected populations (173) underscoring the urgent need for more sophisticated, targeted treatment protocols.

Future strategies to optimize Vitamin D_3_’s therapeutic potential encompass several promising avenues (**Table 5**). Precision medicine approaches, such as integrating VDR genotype analysis with pharmacogenomic profiling, could help determine optimal dosing for individual patients. Machine learning algorithms that combine genetic, metabolic, and clinical data may predict which patients will respond to supplementation, maximizing efficacy while avoiding unnecessary treatment. Development of next-generation Vitamin D_3_ analogues that selectively modulate immune functions with reduced calcemic activity could permit higher effective dosing without toxicity; preliminary candidates have shown the ability to suppress experimental autoimmunity with far less hypercalcemia than calcitriol. Combination therapies also show potential — adding Vitamin D_3_ to methotrexate in RA has enhanced efficacy (174).

Moving forward, research priorities should include large, well-designed clinical trials focusing on Vitamin D_3_–deficient populations and using clinically meaningful endpoints rather than surrogate markers. It is worth noting that ethical constraints make true placebo-controlled trials in Vitamin D_3_-deficient populations difficult to conduct, as most trials allow the control group to receive low-dose supplementation (e.g., 400 IU/day), which may attenuate treatment effects and should be considered when interpreting trial results. Disease-specific studies can identify subpopulations that gain maximum benefit from supplementation. Mechanistic investigations into tissue- and cell-specific Vitamin D_3_ responses are critical to advance the field. Emerging areas — such as Vitamin D_3_’s role in post-viral syndromes, prevention of immunotherapy-related adverse events, and modulation of immunosenescence — warrant intensive study. As evidence grows, clinical guidelines may evolve to incorporate routine Vitamin D_3_ screening and targeted supplementation into standard care for select conditions. Ongoing pharmacovigilance is essential, particularly with novel delivery methods and high-dose uses. Given its favorable safety profile and low cost, Vitamin D_3_, when appropriately targeted, could become a cornerstone for both preventive and adjunctive therapy across diverse clinical contexts.

## 9 Conclusion

Vitamin D_3_ has emerged as a critical regulator of immune homeostasis, with therapeutic potential far beyond its traditional role in mineral metabolism. Through coordinated orchestration of innate and adaptive immunity, Vitamin D_3_ maintains an exquisite balance between effective pathogen defense and suppression of pathological inflammation or autoimmune responses. Its pleiotropic molecular actions on key inflammatory pathways (such as NF-κB and NLRP3 inflammasome suppression) and cytoprotective mechanisms (particularly Nrf2-mediated antioxidant defense) provide multiple intervention targets for diverse immune-related diseases.

The dual nature of Vitamin D_3_—both enhancing antimicrobial defense and suppressing excessive inflammation—represents both opportunities and challenges for clinical translation. This context-dependent immunomodulation means that therapeutic effects vary significantly across different autoimmune diseases, as evidenced by the observed efficacy differences between psoriasis (which responds well to topical treatment) and systemic diseases such as SLE and RA (where oral supplementation shows limited effects). However, translating these mechanistic insights into clinical practice faces substantial challenges: determining optimal dosing regimens, managing individual variability driven by genetic factors, and accounting for environment-dependent effects all require careful consideration and systematic investigation.

To fully realize Vitamin D_3_’s therapeutic potential, the field must embrace biomarker-driven precision medicine approaches. This paradigm shift entails stratifying patients based on comprehensive assessments of baseline 25(OH)D levels, genetic polymorphisms in *VDR*, *CYP27B1* and related genes, and disease-specific factors. Such personalized strategies enable individualized dose optimization, ensuring interventions are targeted to populations most likely to derive meaningful clinical benefit.

The development of advanced delivery systems, combined with biomarker-guided precision strategies, represents a critical path forward for unlocking Vitamin D_3_’s full immunotherapeutic potential. Through this integrated approach, Vitamin D_3_ is transforming from a simple nutritional supplement into a sophisticated, multifunctional targeted agent capable of precisely modulating immune responses. This evolution promises improved outcomes across a spectrum of conditions — including infections, autoimmune diseases, and metabolic disorders — positioning Vitamin D_3_ as a cornerstone of future precision immunotherapy. As our understanding of Vitamin D_3_’s complex immunomodulatory mechanisms continues to evolve, its integration into personalized treatment paradigms will likely reshape approaches to immune-mediated diseases, offering new hope for patients who have not responded to conventional therapies.
